# Children and adolescents exposed to maltreatment already exhibit epigenetic patterns suggestive of heightened low-grade inflammation

**DOI:** 10.1192/j.eurpsy.2022.223

**Published:** 2022-09-01

**Authors:** H. Palma-Gudiel, L. Marques Feixa, S. Romero, M. Rapado-Castro, H. Blasco-Fontecilla, I. Zorrilla, M. Martín, Á. Castro Quintas, J.L. Monteserin-Garcia, E. Font, M. Ramirez, D. Moreno, M. Marín-Vila, N. Moreno, E. Binder, L. Fañanas

**Affiliations:** 1 Centre for Biomedical Research Network on Mental Health (CIBERSAM), Instituto De Salut Carlos Iii, Madrid, Spain; 2 University of Barcelona, Evolutionary Biology, Ecology And Environmental Sciences, Barcelona, Spain; 3 Hospital Clínic, Department Of Child And Adolescent Psychiatry And Psychology, Barcelona, Spain; 4 Hospital General Universitario Gregorio Marañón, Department Of Child And Adolescent Psychiatry, Madrid, Spain; 5 Hospital Puerta de Hierro, Department Of Child And Adolescent Psychiatry, Majadahona, Spain; 6 Hospital Santiago Apostol, Department Of Psychiatry, Vitoria-Gasteiz, Spain; 7 Hospital Benito Menni, Unitat De Crisis D’adolescents, Sant Boi, Spain; 8 Galdakao Mental Health Services, Child And Adolescent Mental Health, Galdakao, Spain; 9 Max-Planck Institute of Psychiatry, Translational Research In Psychiatry, Munich, Germany

**Keywords:** childhood maltreatment, chronic low-grade inflammation, epigenetics, DNA methylation

## Abstract

**Introduction:**

Childhood maltreatment (CM) is one of the best described environmental risk factors for developing any psychiatric disorder, while it also confers increased odds for obesity, cardiometabolic disorders and all-cause mortality. Inflammation has been suggested to mediate the widespread clinical effects of CM. Previously, Ligthart et al. (2016) identified a polyepigenetic signature of circulating CRP levels, a measure of chronic low-grade inflammation, that has been reliably associated with a wide array of complex disorders. The study of this biomarker could dilucidate the mechanistic relationship between CM and psychiatric outcomes.

**Objectives:**

Thus, CRP-associated epigenetic modifications were explored regarding proximal exposure to CM.

**Methods:**

Genomic DNA was extracted from peripheral blood mononuclear cells of 157 children and adolescents (7 to 17 years old). Exposure to CM was assessed following the TASSCV criteria. Genome-wide DNA methylation was assessed by means of the EPIC array. Fifty-two out of the 58 original CRP-associated CpG sites surpassed quality control and were included in the analysis. Age, sex, psychopathological status and cell type proportions were included as covariates.

**Results:**

DNA methylation at 12 out of 52 CpG sites (23%) was significantly associated with exposure to CM (p < .05); 8 of these associations survived correction for multiple testing (q < .05).

**Conclusions:**

This is the first study to date to explore the relationship between childhood maltreatment and an epigenetic signature of chronic low-grade inflammation. Our findings underscore the presence of immune dysregulation early after exposure to CM; further studies are needed to assess the long-term clinical implications of this signature in psychiatric patients.

**Disclosure:**

No significant relationships.

